# Improved foraminal expansion and pain scores following placement of novel expandable interlaminar stabilization device for lumbar spinal stenosis

**DOI:** 10.3389/fsurg.2026.1881593

**Published:** 2026-08-18

**Authors:** Robert P. Norton, Ian Koch, Kendra Primavera, Roy Y. Miloh, Alexander R. Vaccaro, Mitchell Ng

**Affiliations:** 1 Florida Spine Associates, Boca Raton, FL, United States; 2 Department of Orthopaedic Surgery, Broward Health, Fort Lauderdale, FL, United States; 3 Rothman Orthopaedic Institute, Thomas Jefferson University Hospital, Philadelphia, PA, United States

**Keywords:** foraminal expansion, indirect decompression, interlaminar stabilization, lumbar spinal stenosis, posterior column distraction

## Abstract

**Study design:**

Retrospective cohort study.

**Objective:**

To evaluate radiographic durability and clinical outcomes following placement of a novel expandable interlaminar stabilization device for single-level lumbar spinal stenosis. Specifically, we aimed to: 1) quantify immediate postoperative posterior column distraction; 2) determine whether foraminal expansion is improved postoperatively without radiographic subsidence; and (3) assess potential improvements in patient-reported pain/clinical outcomes.

**Methods:**

A retrospective review was performed of adult patients undergoing single-level decompression supplemented by an interlaminar device for symptomatic lumbar spinal stenosis by a single surgeon from 2024 to 2025. Radiographic parameters included anterior, middle, and posterior disc height, foraminal height, and segmental lordosis that were measured preoperatively and longitudinally through 12 months. Postoperative changes were assessed relative to preoperative using paired t-tests, with longitudinal trends analyzed by linear mixed-effects modeling. Clinical outcomes were assessed using Visual Analog Scale (VAS) pain scores. Complications and reoperations were recorded, with statistical significance defined as *P* < 0.05.

**Results:**

A total of 33 patients (mean age 72.6 ± 10.0 years) were included. Posterior disc height increased from 0.46 cm to 0.58 cm (mean +0.12 cm, *P* < 0.001), and foraminal height increased from 1.88 cm to 2.13 cm (mean +0.26 cm, *P* < 0.001). There were no significant changes in anterior disc height, middle disc height, or segmental lordosis. Mixed-effects modeling demonstrated no progressive loss of posterior disc height/foraminal expansion through 12 months postoperatively. Mean VAS improved from 6.8 preoperatively to 4.5 at latest follow-up (mean −2.36, *P* < 0.001). 31/33 patients (93.9%) had no perioperative complications; two (6.1%) required hardware removal following unrelated postoperative trauma or spinous process fracture.

**Conclusions:**

Expandable interlaminar stabilization produced immediate posterior column distraction and foraminal expansion improved at 12 months postoperatively. Clinically meaningful pain reduction was observed in this lumbar stenosis cohort. Our findings support the biomechanical durability and clinical efficacy of interlaminar posterior column stabilization as an adjunct to decompression.

## Introduction

Lumbar spinal stenosis remains a common indication for operative intervention, especially in the context of an aging population (1, 2). Decompression of neural elements has been shown to reliably improve symptoms of neurogenic claudication and/or radiculopathy (3). Instrumented fusion can help provide stability when indicated, but is associated with increased operative time, blood loss, perioperative morbidity, and potential adjacent segment degeneration (4). To this end, there is growing interest in posterior column stabilization surgical strategies that may augment decompression, while limiting the morbidity of pedicle screw-based constructs.

Interspinous process devices were developed to provide indirect decompression and distract the posterior column, with early studies demonstrating improvements in pain and function (5–7). However, concerns regarding spinous process fracture, implant migration, and progressive subsidence limited widespread adoption (8, 9). These complications are thought to arise, in part, from concentrated stress across relatively thin cortical spinous processes and limited load-sharing capacity (9, 10).

Interlaminar stabilization devices represent a further evolution of this concept (8, 9, 11). Interlaminar stabilization devices (ISDs) are indicated as an adjunct to decompression in selected patients with lumbar spinal stenosis. These devices provide segmental stabilization through controlled posterior column distraction and restriction of extension while avoiding the need for pedicle screw based fusion constructs (12). In this cohort, the device was used in order to augment decompression and serve as a scaffold for bone grafting to promote arthrodesis and mitigate further disc collapse and degeneration at the treated level. By having the implant engage with the lamina rather than spinous processes, these constructs help distribute forces across a broader surface area of posterior elements, improving mechanical stability (12). Furthermore, expandable interlaminar systems additionally allow for a more controlled distraction after placement *in situ*, helping facilitate predictable posterior column expansion and indirect neural decompression (13). Despite these advantages, longitudinal radiographic data evaluating the durability of posterior distraction and maintenance of foraminal expansion remain limited (11–13).

To this end, the purpose of this study was to evaluate radiographic and clinical outcomes following placement of a novel expandable interlaminar stabilization device for single-level lumbar spinal stenosis. Specifically, we sought to: (1) quantify immediate postoperative posterior column distraction, (2) determine whether foraminal expansion is improved longitudinally without radiographic subsidence, and (3) assess improvements in patient-reported outcomes. Overall, we hypothesized that this interlaminar engagement and posterior column load-sharing would result in sustained distraction and indirect decompression over time.

## Methods

### Study design and patient selection

A retrospective review was conducted of all adult patients undergoing single-level expandable interlaminar stabilization (KeyLift XL,FloSpine, Boca Raton, FL) as supplemental posterior fixation for symptomatic lumbar spinal stenosis from November 2024 to December 2025. All procedures were performed by a single fellowship-trained spine surgeon at a single institution. Inclusion criteria for the study were: patients over age ≥18 years, radiographic confirmation of single-level lumbar spinal stenosis between L1 and S1, persistent neurogenic claudication and/or radiculopathy refractory to nonoperative management, and treatment with decompression and placement of an expandable interlaminar device (KeyLift XL,FloSpine, Boca Raton, FL). Exclusion criteria included patients undergoing multilevel procedures, surgery indicated due to trauma, tumor, or infection, or revision surgery at the index level.

### Operative technique

**Figure 1:**
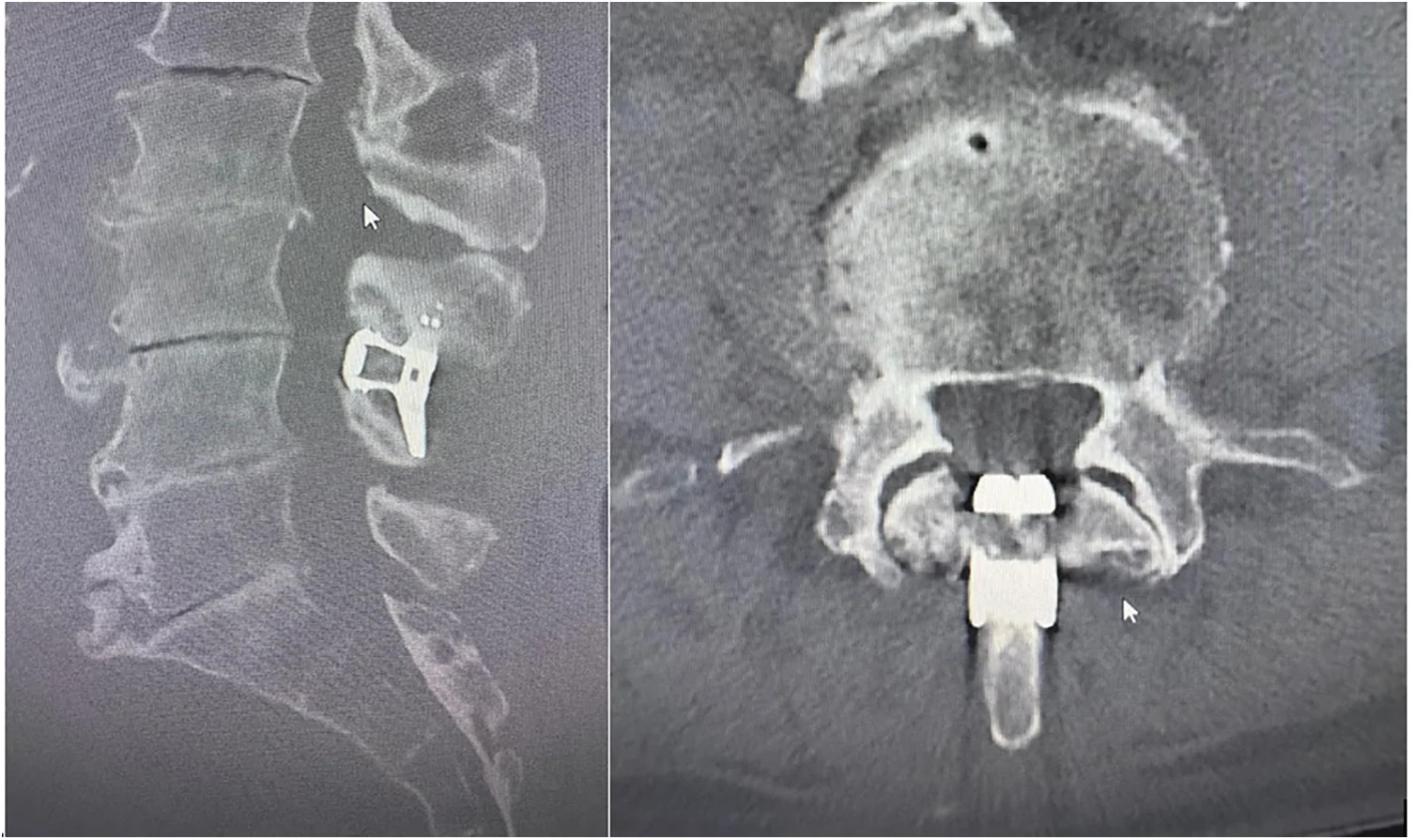
Computed tomography (CT) images demonstrating successful placement of novel interlaminar device.

Through a standard posterior midline approach, lumbar decompression was performed (e.g., laminectomy, partial facetectomy, and/or medial facetectomy) with a motorized burr and Kerrison rongeurs as clinically indicated by the senior surgeon. The interspinous ligament and ligamentum flavum was removed by Leksell rongeur and pituitary rongeurs to allow access to the interlaminar space. Wide decompression of the thecal sac was obtained. Next, a lamina distractor and trial inserters were utilized to determine the appropriate size implant. The expandable interlaminar device (KeyLift XL,FloSpine, Boca Raton, FL) was then inserted between the lamina and expanded/distracted *in-situ* to achieve appropriate distraction of the posterior column (14). The device was used together with supplemental bone graft and fusion. Final implant positioning was confirmed fluoroscopically and on postoperative follow-up, with supplemental bone graft (local autograft and/or allograft) placed to help further promote arthrodesis (Figure 1). Wound closure was performed in standard layered fashion.

### Radiographic assessment

**Figure 2:**
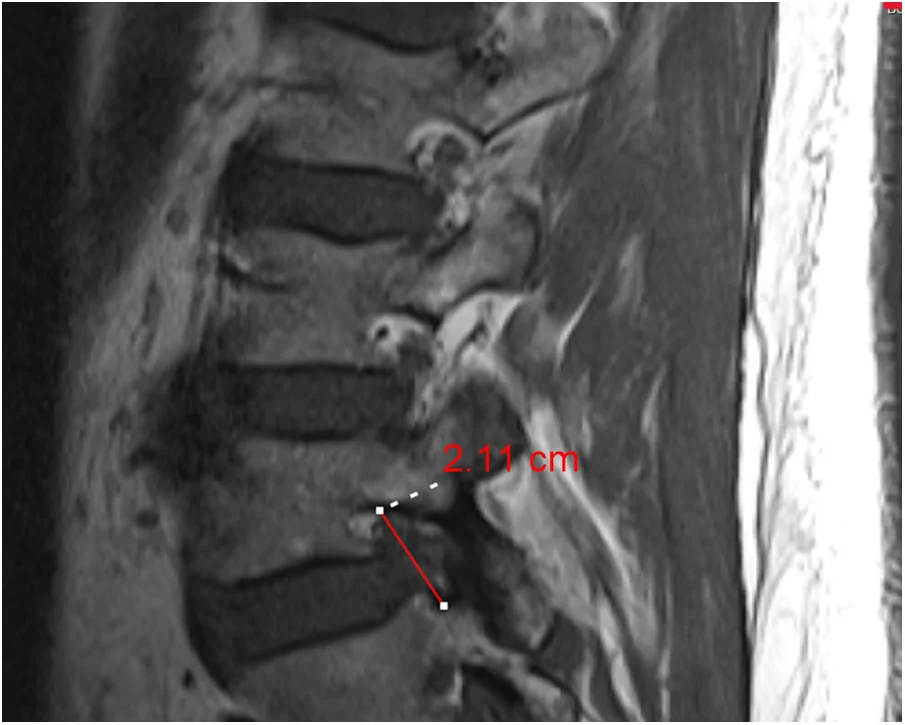
Magnetic resonance imaging (MRI) example measurement of foraminal height.

**Figure 3:**
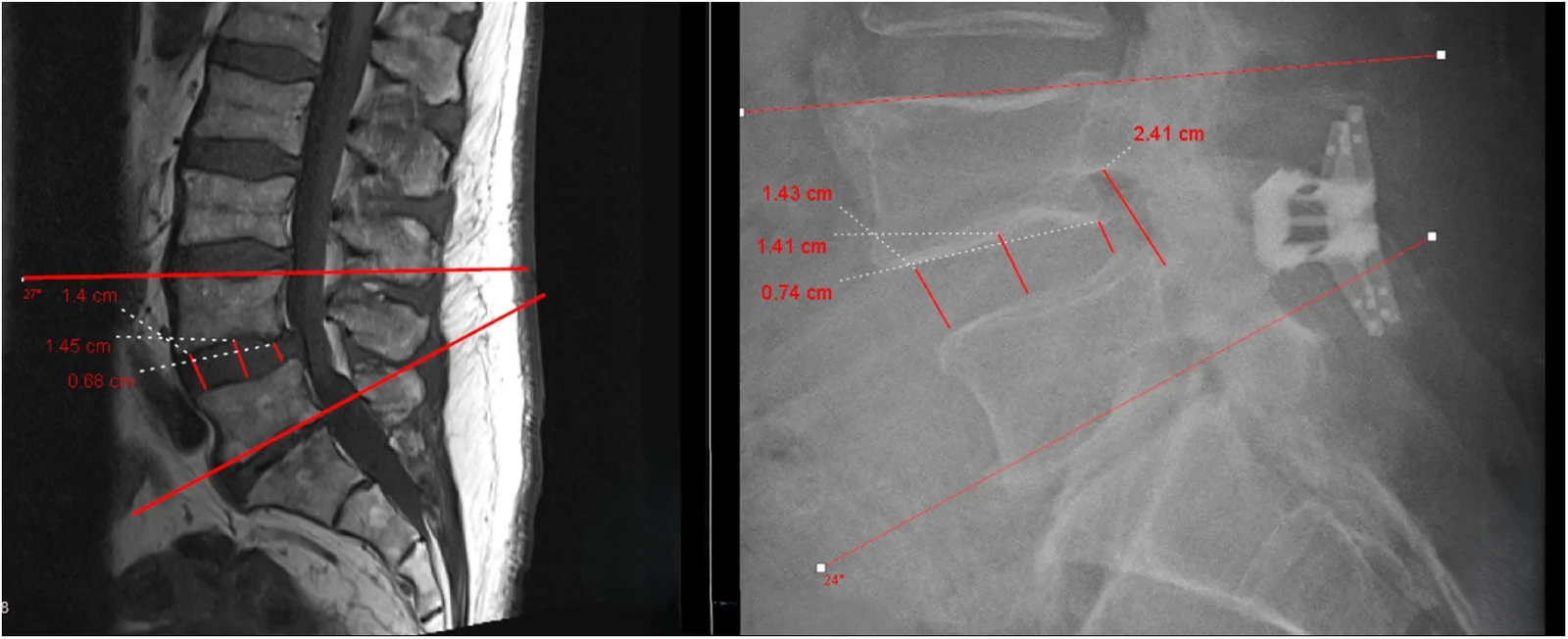
MRI and radiographic example assessment of disc height.

AP and lateral lumbar radiographs were obtained preoperatively and at postoperative intervals specified by the attending surgeon up to 12 months. Radiographic measurements included measuring disc height (anterior, middle and posterior), foraminal height, and segmental lordosis at the treated level. Of note, these radiographic measurements were performed in a standardized format using digital imaging software, referencing vertebral landmarks to ensure consistency (Figures 2, 3). Posterior disc height and foraminal height were prespecified as primary radiographic endpoints, as a marker of posterior distraction and indirect neural decompression. In addition, radiographs were reviewed postoperatively to identify any subsequent implant migration/subsidence, hardware failure, or spinous process fracture.

### Clinical outcomes

Visual Analog Scale (VAS) pain was used to assess and quantify patient reported clinical outcomes. Baseline VAS scores were recorded preoperatively, with postoperative VAS scores obtained at routine follow-up appointments. In addition to numeric overall VAS scores, preoperative documentation included patient-reported distribution of axial vs. radicular pain (percentage symptoms back relative to leg symptoms), allowing characterization of pain predominance patterns. Overall, the primary clinical endpoint was change in VAS from baseline to latest recorded follow-up. Postoperative complications were documented and identified through chart review, including but not limited to new neurologic deficit, wound complications, implant migration, implant failure, spinous process fracture, and reoperation at the index level.

### Statistical analysis

Continuous variables are reported as means and standard deviations, while appropriate categorical variables were reported as frequencies/percentages. Immediate postoperative radiographic changes were assessed using paired t-tests. Linear mixed-effects modeling was used to demonstrate longitudinal radiographic trends, with each patient included as an intercept. This approach allowed detailed inclusion of incomplete follow-up data while appropriately modeling intra-patient correlation and visualization. Regarding patient clinical outcomes, paired analyses were conducted to quantify differences between preoperative and postoperative VAS pain scores, PROMIS Pain Interference and PROMIS Physical Function T-scores. For all statistical analyses, significance was set at *P* < 0.05.

## Results

### Cohort characteristics

A total of 33 patients met inclusion criteria and were included in the final analysis, with a mean age of 72.6 years with a standard deviation of 10.0 years. All underwent single-level interlaminar spacer placement for symptomatic lumbar spinal stenosis. Follow-up extended through 12 months, with variability in intermediate time points typical of retrospective clinical cohorts.

### Successful posterior column distraction and foraminal expansion

**Figure 4:**
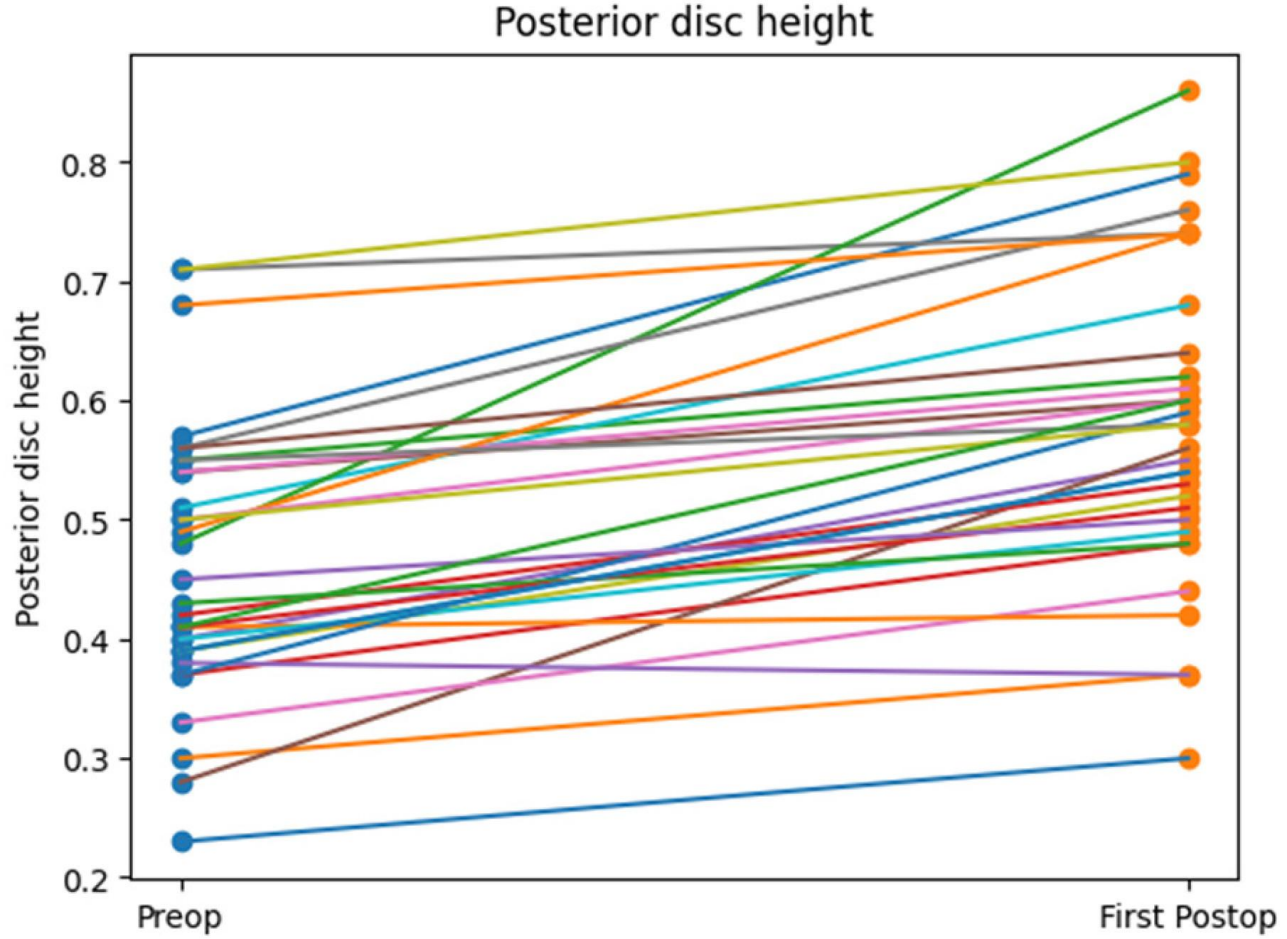
Posterior disc height increased relative to pre-operative measurements.

Comparison of preoperative and early postoperative radiographs demonstrated a statistically significant increase in posterior disc height. Mean posterior disc height increased from 0.46 cm preoperatively to 0.58 cm postoperatively (mean increase 0.12 cm, *p* < 0.001), indicating measurable posterior column distraction at the treated level (Figure 4).

**Figure 5:**
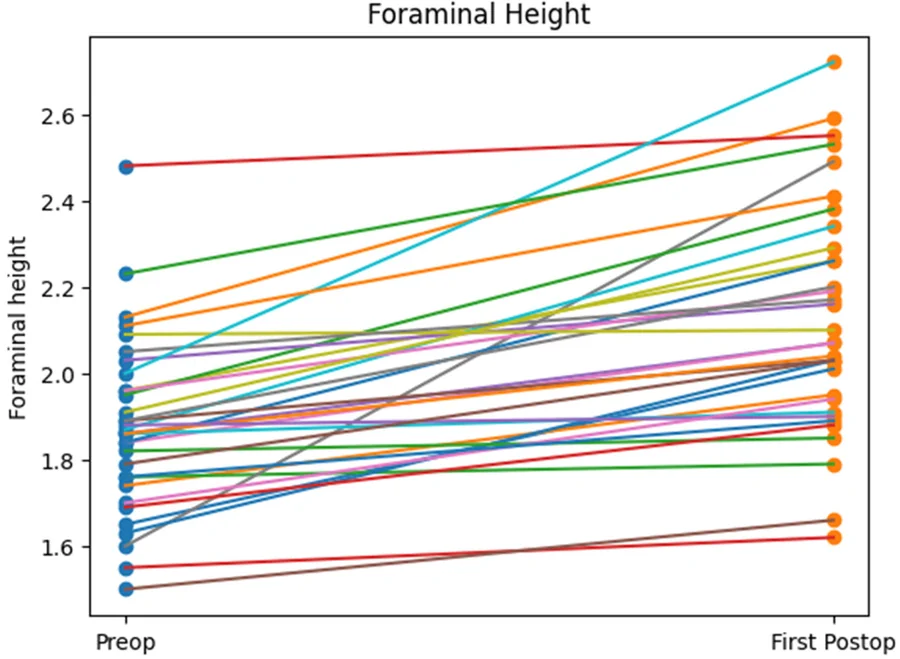
Neuroforaminal height increased relative to pre-operative measurements.

Foraminal height also increased significantly following implantation. Mean foraminal height improved from 1.88 cm to 2.13 cm (mean increase 0.26 cm, *P* < 0.001) (Figure 5), consistent with indirect decompression of the exiting nerve root. No statistically significant changes were observed in anterior disc height, middle disc height, or segmental lordosis.

### Maintenance of radiographic parameters through follow-up

Longitudinal evaluation using linear mixed-effects modeling demonstrated no statistically significant progressive decline in anterior, middle, or posterior disc height over the 12-month follow-up period. The estimated slope of posterior disc height change was approximately zero, with no measurable subsidence. Foraminal height likewise remained stable throughout follow-up without evidence of delayed collapse or loss of distraction. Serial radiographs did not demonstrate progressive implant migration, hardware-related failure, or atraumatic spinous process fracture. One patient underwent a traumatic fall which resulted in an L3-L4 spinous process fracture.

### Early clinical outcomes

**Figure 6:**
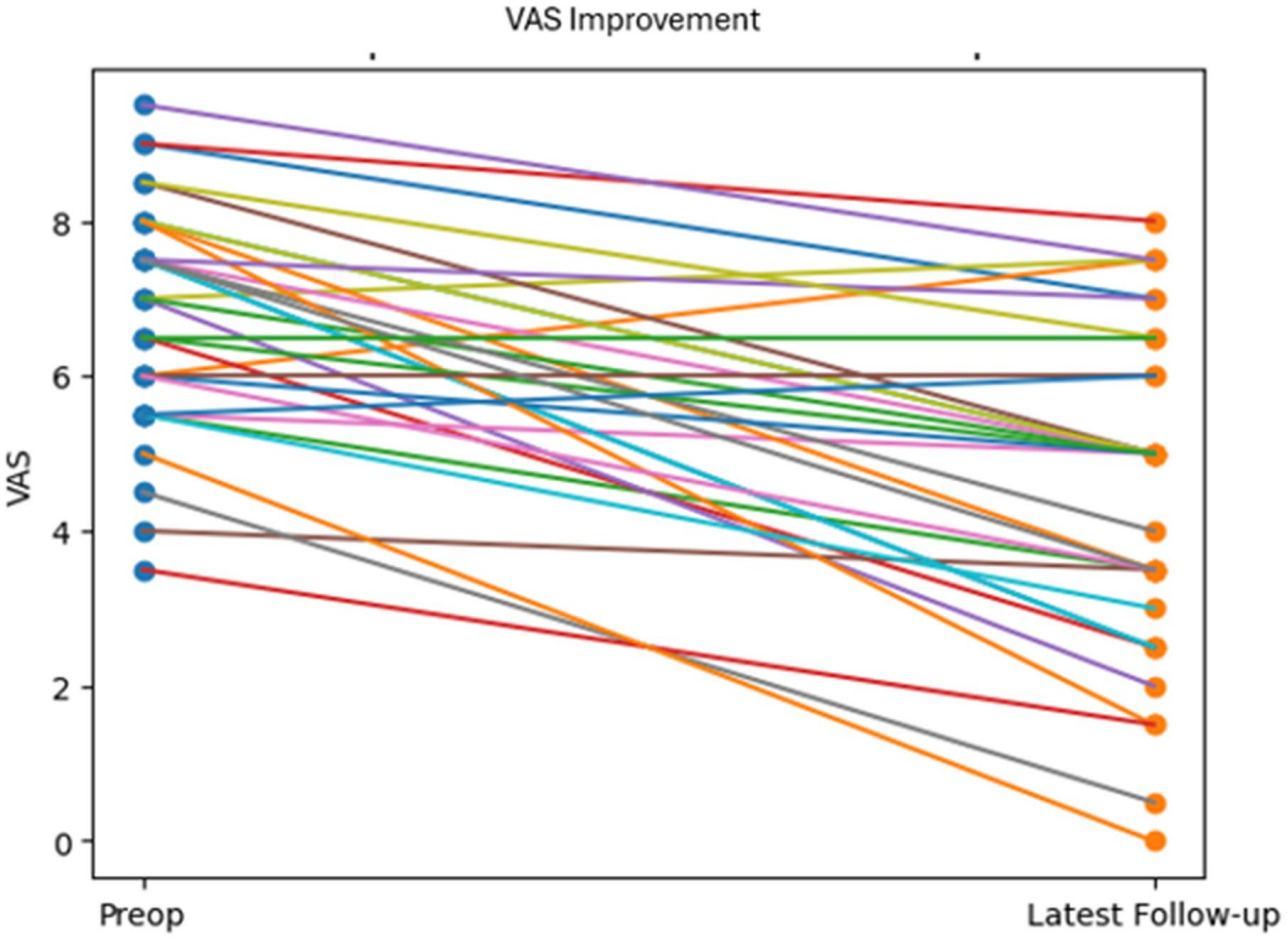
Visual Analog Scores (VAS) pain improvement increased relative to pre-operative.

Preoperatively, 20 patients (60.6%) reported predominantly axial back pain, 9 patients (27.3%) reported predominantly radicular leg pain, and 3 patients (9.1%) reported equal back and leg symptoms. Pain scores improved significantly following surgery (Figure 6). Mean preoperative VAS was 6.8, decreasing to 4.5 at latest follow-up, calculated at approximately the 12-month time point, representing a mean reduction of 2.36 points (*P* < 0.001). This magnitude of improvement exceeds commonly cited minimal clinically important difference thresholds for VAS in lumbar spine surgery.

**Figure 7:**
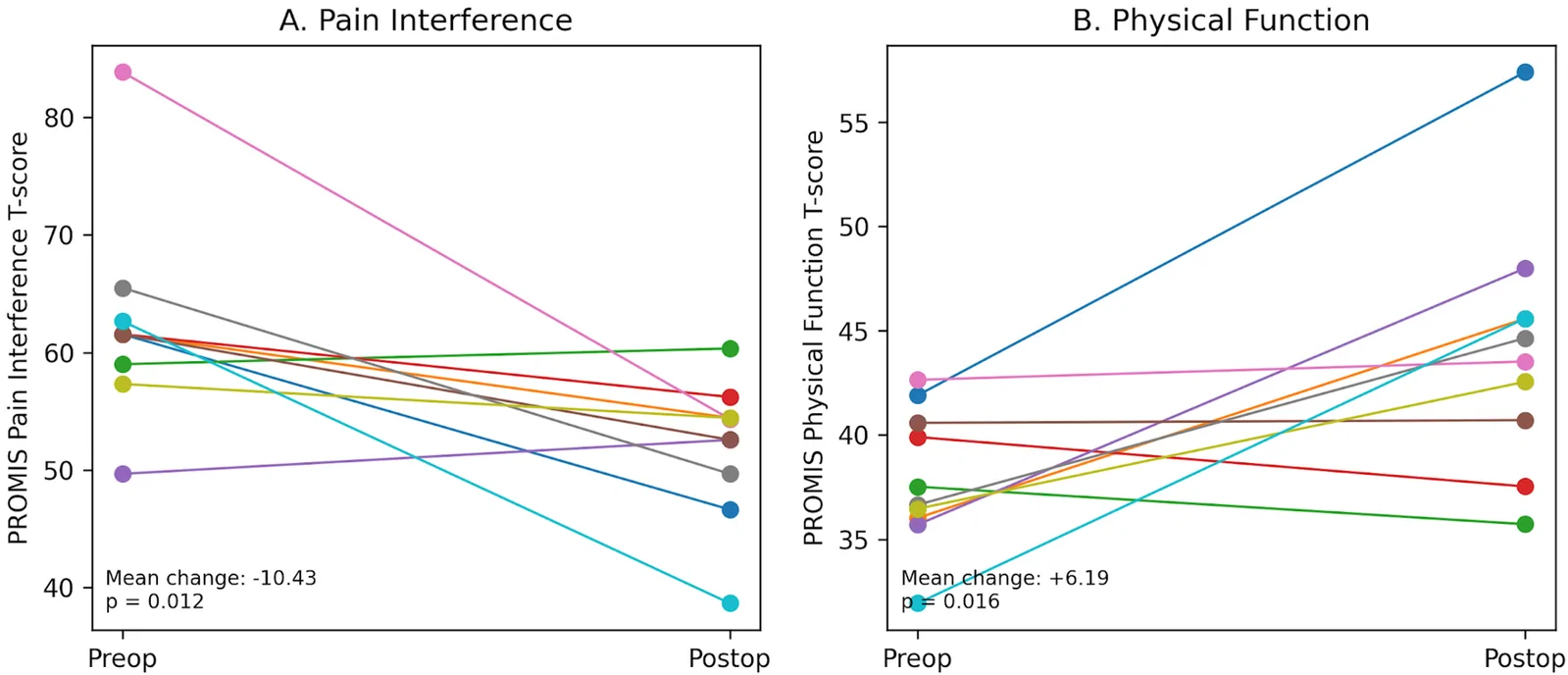
PROMIS pain interference (**A**) and PROMIS physical function (**B**) scores pre-operatively and post-operatively.

Complete paired PROMIS data were available for 10 patients. PROMIS Pain Interference improved from 62.42 ± 8.64 preoperatively to 51.99 ± 5.94 postoperatively, representing a mean change of −10.43 points (95% CI −18.00 to −2.86; *p* = 0.012; Wilcoxon *p* = 0.014; Cohen dz = −0.99). Seven of 10 patients (70%) achieved at least a 5-point improvement in PROMIS Pain Interference. PROMIS Physical Function improved from 37.93 ± 3.29 to 44.12 ± 6.00, corresponding to a mean improvement of 6.19 points (95% CI 1.45 to 10.94; *p* = 0.016; Wilcoxon *p* = 0.037; Cohen dz = 0.93). Six of 10 patients (60%) achieved at least a 5-point improvement in PROMIS Physical Function (Figure 7; Table 1).

**Table 1:** Reported outcomes and *P* values.

Outcome	Pre-op	Post-op	Mean Change	*P*-value
Posterior disc height	0.46 cm	0.58 cm	+0.12 cm	*P* < 0.001
Foraminal height	1.88 cm	2.13 cm	+0.26 cm	*P* < 0.001
VAS pain	6.8	4.5	−2.36	*P* < 0.001
PROMIS Pain Interference	62.42 ± 8.64	51.99 ± 5.94	−10.43	*p* = 0.012 (Wilcoxon *p* = 0.014)
PROMIS Physical Function	37.93 ± 3.29	44.12 ± 6.00	+6.19	*p* = 0.016 (Wilcoxon *p* = 0.037)

Overall, 31 of 33 patients (93.9%) experienced no intraoperative or postoperative complications. Two patients (6.1%) experienced adverse events, including hardware migration following a postoperative fall requiring removal and L3–L4 spinous process fractures with associated hematoma necessitating subsequent hardware removal. No index-level reoperations were performed for device failure or progressive stenosis during the follow-up period.

## Discussion

This study evaluated radiographic durability and clinical outcomes following placement of a novel expandable interlaminar stabilization device for single-level lumbar spinal stenosis. We observed significant increases in posterior disc height and foraminal height following implantation, with no loss of distraction through 12 months postoperatively. Perhaps most importantly, these radiographic findings were accompanied by clinically meaningful reductions in patient-reported pain. Overall, our findings provide radiographic and clinical evidence that such implants can be safely placed without migration/subsidence, while effectively reducing pain.

Our first finding demonstrated a statistically significant distraction of the posterior column, as measured via posterior disc height and foraminal height. Prior investigations of interspinous devices have reported variable radiographic expansion following implantation, with some studies suggesting limited or inconsistent restoration of foraminal dimensions. One retrospective study of 101 interspinous devices placed found a significant decrease in mean anterior disc height (ADH) and range of motion after surgery, with 14% of patients experiencing erosion defined as a radiolucent gap between spinous process and device (15). In contrast, expandable interlaminar systems allow controlled *in situ* distraction, potentially enabling more consistent and predictable distraction of the posterior column (13). The magnitude of distraction observed in our cohort, though modest in absolute terms, was consistent across all patients. These findings align with biomechanical studies suggesting that laminar engagement may facilitate controlled posterior distraction while distributing load more evenly across posterior elements (11, 13).

Regarding the durability of these radiographic improvements, our results demonstrate maintenance of posterior disc height and neuroforaminal expansion without radiographic evidence of progressive subsidence up to 12 months. Historical experience with interspinous devices has identified concerns regarding delayed loss of distraction, migration, and structural failure, often attributed to stress concentration across the spinous processes (5, 8, 9). A robust study of 1,108 patients undergoing interspinous device found an overall failure rate of 9.6%, including a 7.8% complication rate (27 spinous process fractures, 23 dural tears with cerebrospinal fluid leakage) (16). Reasons for revision always involved removal of original implant due to acute worsening of low back pain (45 cases), lack of improvement, symptom recurrence (42 cases), and implant dislocation (20 cases) (16). The absence of measurable collapse in our cohort may reflect the broader cortical engagement and load-sharing characteristics of interlaminar constructs relative to interspinous devices. While the follow-up period remains limited to one year, the stability of radiographic parameters across serial assessments suggests mechanical equilibrium rather than gradual implant settling.

Clinically, we observed a significant reduction in overall VAS pain scores. The MCID thresholds for lumbar spine surgery are commonly cited as a 30% reduction in pain and/or absolute improvement of 2.5–3.5 points on a 10-point scale (17, 18). The mean reduction in VAS pain score of 2.36 points, from 6.8 preoperatively to 4.5 at final follow-up, represents a statistically significant improvement. While this change did not exceed the commonly cited absolute MCID threshold of 2.5–3.5 points for VAS pain, it did meet the 30% relative-change MCID criterion, suggesting a clinically meaningful benefit for many patients. This warrants careful consideration when interpreting treatment effect magnitude. The residual mean VAS score of 4.5 indicates that patients continued to experience a moderate degree of pain postoperatively. Therefore, it is key to remember that symptom improvement is not synonymous with complete pain resolution.

We also observed a significant reduction in PROMIS Pain Interference scores (Wilcoxon *p* = 0.014), and improvement in PROMIS Physical Function scores (Wilcoxon 0.037). The PROMIS findings support the clinical relevance of the radiographic observations. Despite the limited availability of PROMIS follow-up data, patients with paired measurements demonstrated statistically significant and clinically meaningful improvement in both pain interference and physical function. PROMIS data were substantially more incomplete than VAS data, and the PROMIS results should therefore be interpreted as supportive rather than definitive. Prospective PROMIS collection at standardized intervals would strengthen future analyses and allow more robust longitudinal modeling. Of note, preoperatively our cohort included a heterogeneous mix of patients with either axial-predominant and/or radicular-predominant pain patterns (19, 20). The indirect decompression achieved through posterior column distraction may potentially contribute to symptomatic improvement by enlarging foraminal dimensions and reducing dynamic nerve root compression (21, 22). Although decompression itself remains the principal driver of neurologic symptom relief, adjunctive posterior stabilization may in theory also provide benefit in patients by limiting unintended motion and improving intervertebral neuroforaminal height (23). Importantly, our overall observed complication rates were low, with no index-level reoperations for device failure or recurrent stenosis during follow-up.

Several studies have evaluated interlaminar stabilization as an adjunct to decompression for lumbar spinal stenosis, providing additional context for the findings of this study. A prospective, randomized FDA Investigational Device Exemption trial compared decompression with Coflex interlaminar stabilization to decompression with instrumented posterolateral fusion. At 2 and 5 years of follow up, patients undergoing interlaminar stabilization achieved clinical improvements in Oswestry Disability Index (ODI), visual analog scale (VAS) pain scores, and Zurich Claudication Questionnaire outcomes that were comparable to those observed after fusion. Radiographic analyses at 2 years demonstrated significantly less angulation at the superior adjacent level in the interlaminar stabilization cohort, which may suggest less biomechanical stress on adjacent segments. Although reoperation rates were numerically higher in the interlaminar stabilization group at 2 years, the difference was not statistically significant (24). Evidence directly comparing decompression with and without interlaminar stabilization remains limited. Stand-alone laminectomy remains an effective treatment decision for patients that are selected appropriately (25). However, postoperative instability, progressive disc collapse, and recurrent stenosis may occur at a higher rate in patients that require more extensive decompression or in those with pre-existing degenerative instability (26). In these patients, interlaminar stabilization can provide posterior column support and avoid the morbidity associated with pedicle screw instrumentation. This may represent an intermediate treatment option between decompression alone and complete fusion (27).

There are several potential limitations to this study. First, this study is retrospective in design and represents the experience of a single surgeon at a single institution, which may limit external validity and overall generalizability. Second, with an overall modest sample size with limited follow-up to 12 months, we are unable to draw conclusions on long-term outcomes. In addition, radiographic measurements were derived from plain lateral radiographs rather than cross-sectional CT imaging, which may introduce measurement variability. Although pain outcomes improved, we did not assess functional outcomes data for this cohort. Furthermore, the lack of a control group of patients who underwent decompression alone or with traditional instrumented fusion limits our ability to compare outcomes across various operative techniques. Of note, radiographic measures were performed by a Physician assistant and a resident, with any disagreements resolved by consensus discussion. Measurements were not blinded and therefore this should be noted as a limitation in this study. Another limitation of this study is that this was an early, single-surgeon experience with a novel interlaminar stabilization device. The absence of a comparator group is a limitation, as this would provide a better benchmark to compare outcomes. Future studies should be created with better comparison between these two groups. Future prospective, comparative studies with longer-term follow-up will be necessary to further define the role of expandable interlaminar stabilization in the treatment algorithm for lumbar spinal stenosis. Nevertheless, our study is the first to report the safety and efficacy of novel expandable lumbar interlaminar devices implanted with excellent radiographic and clinical outcomes.

## Conclusion

In this retrospective cohort of 33 patients, we found that expandable interlaminar stabilization following single-level decompression resulted in immediate posterior column distraction and foraminal expansion that were improved at 12 months without radiographic evidence of subsidence. Our radiographic findings were followed by clinically meaningful reductions in pain and an overall low complication profile. Altogether, our data demonstrates the short-term safety and clinical efficacy of expandable interlaminar posterior column stabilization to augment decompression in appropriately selected patients with lumbar spinal stenosis.

## Data Availability

The raw data supporting the conclusions of this article will be made available by the authors, without undue reservation.
